# Differential Gene Expression in High- and Low-Active Inbred Mice

**DOI:** 10.1155/2014/361048

**Published:** 2014-01-16

**Authors:** Michelle Dawes, Trudy Moore-Harrison, Alicia T. Hamilton, Tyrone Ceaser, Kelli J. Kochan, Penny K. Riggs, J. Timothy Lightfoot

**Affiliations:** ^1^Department of Health and Kinesiology, Texas A&M University, College Station, TX 77843, USA; ^2^Sydney and JL Huffines Institute for Sports Medicine and Human Performance, Texas A&M University, College Station, TX 77843, USA; ^3^Department of Kinesiology, University of North Carolina Charlotte, Charlotte, NC 28223, USA; ^4^Department of Animal Science, Texas A&M University, College Station, TX 77843, USA

## Abstract

Numerous candidate genes have been suggested in the recent literature with proposed roles in regulation of voluntary physical activity, with little evidence of these genes' functional roles. This study compared the haplotype structure and expression profile in skeletal muscle and brain of inherently high- (C57L/J) and low- (C3H/HeJ) active mice. Expression of nine candidate genes [*Actn2*, *Actn3*, *Casq1*, *Drd2*, *Lepr*, *Mc4r*, *Mstn*, *Papss2*, and *Glut4* (a.k.a. *Slc2a4*)] was evaluated via RT-qPCR. SNPs were observed in regions of *Actn2*, *Casq1*, *Drd2*, *Lepr*, and *Papss2*; however, no SNPs were located in coding sequences or associated with any known regulatory sequences. In mice exposed to a running wheel, *Casq1* (*P* = 0.0003) and *Mstn* (*P* = 0.002) transcript levels in the soleus were higher in the low-active mice. However, when these genes were evaluated in naïve animals, differential expression was not observed, demonstrating a training effect. Among naïve mice, no genes in either tissue exhibited differential expression between strains. Considering that no obvious SNP mechanisms were determined or differential expression was observed, our results indicate that genomic structural variation or gene expression data alone is not adequate to establish any of these genes' candidacy or causality in relation to regulation of physical activity.

## 1. Introduction

The benefits of physical activity on health and disease have been demonstrated convincingly [1]. Despite this evidence, physical activity continues to decline in humans [2, 3], with data suggesting that less than 5% of adults complete moderate activity on a regular basis and 25% of adults are not active at all during their leisure time. Physical inactivity is a risk factor for many health outcomes such as cardiovascular disease, diabetes, some forms of cancer, and obesity [4].

Studies of both human and animal models strongly suggest that genetic factors play a role in physical activity with little common environmental effect [5–15]. Heritability of physical activity has been observed to widely range from 20% to 92% in humans and mice, depending on the heritability index used, the activity measurement employed, the sex and age of the subject, and species, among other factors. While copious evidence exists that genetics are associated with the determination of physical activity levels, little direct evidence supports involvement of specific genetic mechanisms in activity regulation.

Recently, several putative candidate genes have been proposed to play roles in physical activity; however, there has been no definite consensus about what constitutes “sufficient evidence” to define a candidate gene. Traditional experimental approaches most often have used the single criterion of functional relevance as the standard for candidate gene declaration [16]. DiPetrillo et al. [17] suggested that a candidate gene can be declared when a potential candidate gene exhibits at least three lines of evidence as to its involvement in the phenotype of study, which includes location within a known QTL, differences in gene expression, the aforementioned “functional relevance,” and/or alteration in the phenotype with manipulation of the gene. An example of this approach can be seen with two candidate genes for physical activity—dopamine receptor 1 (*Drd1*) and nescient helix loop helix 2 (*Nhlh2*)—which have shown functional relevance to activity [18, 19], interval-specific haplotype differences in animals exhibiting differential phenotypes [20], localization within identified activity single-effect and epistatic QTL [10, 12, 21–24], expression differences between high- and low-active animals [25], and/or a change in phenotype with gene manipulation [18, 26]. However, unlike *Drd1* and *Nhlh2*, the majority of potential candidate genes suggested to be associated with physical activity have little evidence to support their candidacy [27].

Therefore, the purpose of this study was to examine the interval-specific haplotype structure and gene expression of the nine previously suggested [10–12, 27], but weakly supported, candidate genes in high- (C57L/J) and low-active (C3H/HeJ) mice in both central brain (nucleus accumbens) and peripheral musculoskeletal (soleus) tissues, with the goal of adding additional lines of evidence to support these genes as candidates for future causal activity regulation studies.

## 2. Methods

### 2.1. Overall Procedures

Based on the available literature, nine genes with direct or indirect association (through functional relevance or GWAS) to physical activity were investigated: actinin 2 (*Actn2*, [27]), actinin 3 (*Actn3*, [27]), calsequestrin 1 (*Casq1*, [28]), dopamine receptor 2 (*Drd2*, [29]), leptin receptor (*Lepr*, [13, 30]), melanocortin 4 receptor (*Mc4r*, [31]), myostatin (*Mstn*, [27]), 3′-phosphoadenosine 5′-phosphosulfate synthase 2 (*Papss2*, [32]), and glucose transporter 4 (*Glut4*—aka *Slc2a4*, [33]). Two methods were used to investigate these genes. Initially, published databases were interrogated to identify regional haplotype differences indicating potential genomic variation in the candidate gene between the high- and low-active mouse strains. Second, mRNA expression was measured in both naïve and running wheel-exposed mice of both strains.

### 2.2. Method One: Interval-Specific Haplotype Comparisons

Haplotypes of the nine candidate genes were compared within and between the high- and low-active mouse strains to identify potential genetic structural differences that could contribute to phenotypic variation. Initial haplotype analysis was conducted using the dense single nucleotide polymorphism (SNP) map from Perlegen Inc. (Mountain View, CA) (*≈*8.3 million SNPs). The Perlegen database utilized sequence data from 55 inbred strains of mice to predict haplotypes using pairwise comparisons between mouse strains. The specific chromosomal location of each target gene was determined using the NCBI GENE database (http://www.ncbi.nlm.nih.gov/gene/) and then inserted into the haplotype block viewer [20]. The haplotype viewer provided a binary determination of whether the haplotype and any SNPs, if present, were similar or dissimilar between the strains. Following the recent dismantling of the Perlegen online mouse haplotype viewer, the haplotype data were subsequently reverified using the Mouse Phylogeny Viewer (http://msub.csbio.unc.edu/ [34]).

### 2.3. Method Two: Gene Expression Determination

We had previously identified C3H/HeJ inbred mice as low-active and C57L/J inbred mice as high-active [10] with the C57L/J mice running, on average, 271% farther on a daily basis than the low-active C3H/HeJ mice. At eight weeks of age, four C57L/J and four C3H/HeJ mice (Jackson Labs, Bar Harbor, ME) were housed individually in cages with a 450 mm circumference solid surface running wheel (Ware Manufacturing, Phoenix, AZ) interfaced with a magnetic sensor and computer odometer (Sigma Sport BC600, St. Charles, IL) that counted revolutions of the running wheel and total time the mouse ran. Each cage computer was calibrated (as per manufacturer's instructions) for the circumference of the cage wheel allowing for measurement of distance (km) and time (min) the animals ran on the wheel, with subsequent calculation of speed (m/min). After one week of adaptation to the wheel, the activity of each mouse was monitored every 24 hours beginning at 63 days of age (9 weeks) for seven consecutive days. Each day the wheels were checked to insure that they turned freely. These methods have been validated for repeatability [35]. Subsequently, due to concerns that wheel exposure would cause training-induced gene expression changes, a separate group of high-active and low-active mice (*n* = 12, 3 ♂ and 3 ♀ of each strain) were housed with locked (i.e., nonturning) wheels from 8 to 10 weeks of age. Mice of respective activity groups were housed in the same room of the university vivarium with 12 h light/dark cycles (see discussion), with temperature and humidity maintained at 19–21°C and 50–60%, respectively. Food (Harland Tekland 8604 Rodent Diet, Madison, WI) and water were provided *ad libitum*. Mice were weighed on a weekly basis. At 10 weeks of age, the mice were anesthetized with 2–4% isofluorine for body composition testing and subsequently euthanized. The nucleus accumbens and the soleus muscle were harvested and flash frozen in liquid nitrogen and then stored at −80°C for later analysis. Body composition was analyzed in the naïve animals prior to tissue harvesting, using the Lunar Piximus DEXA (dual-energy X-ray absorptiometry) instrument (Fitchberg, WI). All procedures were approved by the University of North Carolina Charlotte and Texas A&M University Institutional Animal Care and Use Committees.

Target gene transcript expression was measured by quantitative real-time polymerase chain reaction (RT-qPCR) as reported previously, with minor modifications [25]. Total RNA was isolated from nucleus accumbens and soleus tissue using the Qiagen RNeasy mini kit (Qiagen, Valencia, CA). Immediately following the elution step, DNA was removed with a DNA-*free* kit (Ambion, Austin, TX). RNA was quantified using a NanoDrop 1000 spectrophotometer (Thermo Scientific, Waltham, MA) and in naïve animals quality of RNA was determined by an Agilent 2100 Bioanalyzer (Santa Clara, CA). RNA samples with RIN quality values >7.5 were included in RT-qPCR assays. RNA was reverse transcribed using iScript Reverse Transcription Supermix for RT-PCR (Bio-Rad Laboratories, Hercules, CA). Then RT-qPCR was conducted using SsoFast Probes Supermix with ROX (Bio-Rad Laboratories), along with predesigned PrimeTime RT-qPCR Assays (Integrated DNA Technologies, (IDT), Coralville, IA) and 2 *μ*L cDNA to detect the transcript sequence of interest. All reactions were run in duplicate. RT-qPCR reactions were run on an Applied Biosystems 7900HT Fast Real-Time PCR System (Carlsbad, CA). A fivefold RNA dilution series was utilized to determine efficiency of each qPCR assay. Amplification data were analyzed with Sequence Detection Software v. 2.2.2 (Applied Biosystems). Expression was normalized to an endogenous control (18S ribosomal RNA (*RN18S*; IDT)) using methods described by Pfaffl [36]. A gene expression ratio was calculated that is positively related to expression level and takes the efficiency of each assay into consideration. Briefly, gene expression ratio (GER) = target gene efficiency^(CT  target  reference  −  CT  target  gene)^/control gene efficiency^(CT  control  reference  −  CT  control  gene)^. The reference value (calibrator) used for a given gene was the average Ct of all samples (in both strains) for that gene. Efficiency was calculated using the slope of the standard curve (10^(−1/slope)^).

*Actn2*, *Casq1*, *Glut4*, *Lepr*, and *Mstn* expression levels were measured in both the nucleus accumbens (central) and soleus (peripheral) tissue of animals exposed to running wheels. Based on evidence in the literature and results from the wheel-exposed animals, expressions of *Actn3*, *Actn2*, *Casq1*, *Glut4*, *Lepr*, and* Mstn* were assayed in the soleus of the naïve animals, while *Drd2*, *Mc4r*, *Papss2*, and *Lepr* were measured in the nucleus accumbens of the naïve animals.

### 2.4. Statistics

Gene expression data were checked for normality using a two-sided *F* test (JMP 10.0, SAS Institute, Cary, NC). If the expression ratio was not normal (*P* < 0.05), the expression data were analyzed by Chi-square nonparametric approaches. Normally distributed expression ratios were compared by a pooled *t*-test (if variances were equal) or Student's *t*-test (if variances were not equal). Alpha values were set *a priori* at 0.05. In all analyses, expression values that were greater than 2.5 standard deviations away from the mean were considered outliers and eliminated from the dataset. If differential expression was observed, data were subsequently analyzed for sex differences.

## 3. Results

No difference in body weight was observed between strains for mice exposed to the running wheel (23.7 g ± 2.6 g C57L/J versus 23.3 g ± 3.6 g C3H/HeJ, mean ± SD; *P* = 0.93) or naïve animals (24.0 g ± 2.6 g C57L/J versus 24.4 g ± 2.1 g C3H/HeJ; *P* = 0.43). In naïve animals, percent body fat was not different between the strains (12.7% ± 1.6% C57L/J versus 14.5% ± 1.8% C3H/HeJ; *P* = 0.09).

**Figure 1:**
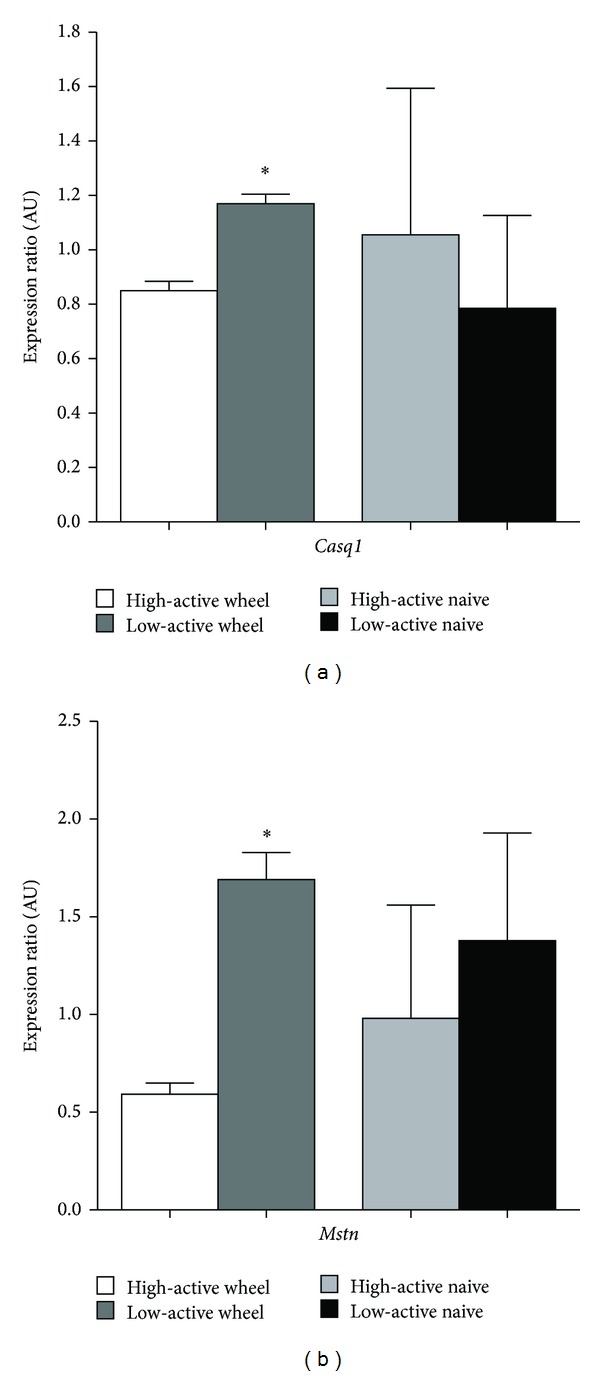
Expression of *Casq1* (a) and *Mstn* (b) in soleus muscle. In both panels, comparisons made between strains within activity state (high-active versus low-active). *Significantly different from wheel-exposed high-active mice (*P* < 0.05). Values are mean ± SD. AU, arbitrary units.

Differential haplotypes were exhibited across the entire transcribed region of *Actn2*, *Casq1*, *Drd2*, *Lepr*, and *Papss2*, as reflected by a number of SNPs in each gene (Table 1). The strains exhibited similar haplotype patterns for *Mstn*, *Glut4*, *Mc4r*, and *Actn3* (i.e., no differential SNPs). *Casq1* (*P* = 0.0003) and *Mstn* (*P* = 0.002) transcript expression in the soleus was found to be different between the high-and low-active mice exposed to a running wheel (Table 2; Figure 1), while there were no differences observed in *Actn2* (*P* = 0.55), *Glut4* (*P* = 0.20), or *Lepr* (*P* = 0.85). However, when these genes were evaluated in the soleus between strains of naïve animals, differences in expression of *Casq1* and *Mstn* were not observed (*P* = 0.40 and *P* = 0.27, resp.). No differential expression was observed in any of the genes evaluated in the nucleus accumbens (*Actn2*, *P* = 0.13;* Casq1*, *P* = 0.64; *Glut4*, *P* = 0.58; *Lepr*, *P* = 0.72; *Mstn*, *P* = 0.37; Table 2) in animals exposed to the running wheels.

In naïve animals, gene expression results indicated no differential expression between high- and low-active animals for any of the genes in the soleus (*Actn2*, *P* = 0.58; *Actn3*, *P* = 0.58; *Casq1*, *P* = 0.40;* Glut4*, *P* = 0.22; *Lepr*, *P* = 0.82; *Mstn*, *P* = 0.27; Table 2). No difference was seen between strains in *Drd2* (*P* = 0.06), *Lepr* (*P* = 0.18), *Mc4r* (*P* = 0.08), or *Papss2* (*P* = 0.40) in the nucleus accumbens (Table 2). Gene expression differences between sexes were not observed in either strain.

## 4. Discussion

As an extension of quantitative genetic approaches that have been used to investigate the genetic control of physical activity, several genes have been suggested to be associated with activity regulation with little or no supporting physiological evidence for their involvement. This study's purpose was to investigate whether nine putative candidate genes had interval-specific haplotype structure variability and were actually expressed differentially between high- and low-active mice. Although differential gene expression is not the only determinant of whether a gene is a candidate gene, it is one line of evidence suggesting that a gene may be involved in regulation of a particular phenotype. We found that prior exposure to a running wheel, in and of itself, caused changes in gene expression, demonstrating a training effect. Thus, as our goal was to investigate innate differences in gene expression between strains with varying activity levels, expression was subsequently measured in naïve mice. Interestingly, although these strains of mice have distinctively diverse levels of activity, none of the genes evaluated were differentially expressed between naïve high- and low-active mice in the nucleus accumbens or soleus. The majority of genes evaluated in this study were chosen from genome-wide association studies utilizing genomic DNA, which does not correspond to transcript levels. Therefore, differential expression between phenotypes should not necessarily be expected from genotype association studies alone. While not ruling these genes out as potential regulators of physical activity, our data provides evidence that differences in activity are not due to variability in transcript abundance in this model. Likewise, given that there are no SNPs located in protein-coding regions for any of the genes evaluated genomic variability between the strains in these genes does not account for phenotypic differences between strains. Thus, while association and functional relevance provide two lines of evidence, we suggest that further functional validation of these genes is necessary, possibly including investigation of post-transcriptional modification and differences in regulatory mechanisms as additional lines of support for the gene's candidacy in relation to any phenotype regulation.

It has been well established that genetic background is a significant regulator of daily physical activity in both humans and mice, with little input from common environmental influences [5–15, 23, 37–39]. In spite of the mounting evidence confirming genetic control of physical activity, little is known about the actual regulatory mechanisms, including the identity of the responsible genes. Identification of potential candidate genes has been primarily through speculated functional relevance and/or location within an identified quantitative trait locus, with little or no functional validation. More often than not, further examination of potential candidate genes has indicated that use of QTL location/perceived physiological relevance results in a large number of false positive quantitative trait genes (QTG). Indeed, the early promise of discovering QTG from QTL has had limited success, with some authors reporting less than a 1% success rate in finding QTG in QTL [16]. Flint et al. [16] also suggest that candidate genes derived from most QTL studies account for very small phenotypic effects. Therefore, the small effects of putative candidate genes associated with QTL, combined with sequence variance and position of the QTL relative to the coding region of the gene, make determining the actual causative gene and function using traditional quantitative genetic approaches extremely difficult.

For example, De Moor et al. [32] found novel SNPs in the *Papss2* gene region related to activity levels in humans, suggesting *Papss2* was associated with leisure time exercise behavior. *Papss2* produces a sulfonation enzyme that modifies macronutrients and exogenous compounds and is expressed in many tissues including skeletal muscle and brain [32]. In our mouse model, however, we found no differences in *Papss2* expression between our high- and low-active mice in the nucleus accumbens, a region of the brain that has been suggested as a primary site of activity regulation [25, 40]. *Papss2* was not expressed at observable amounts in the soleus of our mice using the methods employed, although this may have been due to the small quantities of RNA available to use in the reverse transcription reaction. Interestingly, all of the SNPs found in De Moor's work were located in intron 1 of *Papss2*. Likewise, we found five SNPs in intron 1 of *Papss2* between our strains of mice (Table 1); however, a BLAST comparison of the human and mouse gene sequence shows that none of the SNPs identified seem to match between species. Intronic SNPs are spliced out of the mRNA, therefore not affecting sequence of the mature transcript. Intronic sequence variance would only impact transcript levels through alteration of miRNA sequences or by location in the promoter region. None of these modes of regulation are currently presented in the literature for *Papss2*. As DNA sequence variation does not have a causal relationship with transcript abundance, we should not be surprised that our results differ from those of De Moor's et al. [32].

Unraveling the regulatory mechanisms of voluntary activity is further complicated by a variety of genetic mechanisms contributing to transcriptional regulation. Therefore, it is critical that potential candidate genes be examined thoroughly before they become entrenched in the literature as “causative” of a phenotype. With only 2% of the human genome actually coding for proteins, it is not surprising that mechanisms other than structural gene variation contribute to differences in phenotype. Regulatory regions of noncoding sequences may be contributing to regulation of voluntary physical activity through a variety of mechanisms (e.g., miRNA, siRNA, and ribosomal binding proteins [41]). These regulatory mechanisms have not been fully characterized and may be contributing to activity regulation as we have previously suggested [10]. We have shown that other genetic mechanisms such as epistasis (gene interactions) and pleiotropy (one gene has multiple effects) can affect physical activity regulation [23, 42, 43]. *Glut4* was selected as a putative candidate gene for inherent physical activity regulation based on QTL association [21, 23, 27] and from functional relevance [33]. *Glut4* functions to move glucose across the plasma membrane of cells, is found in skeletal muscle, and is induced by insulin or exercise [33]. Tsao and colleges [33] observed that mice with *Glut4* overexpression ran four times further than controls. *Glut4* was found to be close to the “mini-muscle” gene region [21] as well as near a QTL exhibiting significant epistasis for distance run [23]. Considering these previous physical and functional experiments of the role of *Glut4* in physical activity, we expected to see differential expression between our inherently high- and low-active strains of mice. However, like *Papss2*, we observed no differences in expression. It is possible that* Glut4* may function through epistasis with other genes; thus differential expression of *Glut4* itself would not be detected.

Considering the multitude of mechanisms contributing to gene regulation, it is not unreasonable that the only differential gene expression observed between strains in this study was due to a training effect. It is well known that a variety of perturbations can influence gene expression, such as repeated exercise bouts altering transcript levels in skeletal muscle and brain tissue [44, 45]. While we had not previously shown alteration in brain gene expression after running wheel activity [25], it is not surprising that even a minimal exposure to wheel running (seven days) produced changes in some of the skeletal muscle genes measured (*Mstn* and *Casq1*). The literature is ambiguous for *Mstn* expression changes in skeletal muscle with endurance exercise training, showing variable results depending on species, training mode, and time elapsed after exercise session, amongst other factors [46, 47]. *Casq1* protein levels have been shown to decrease in the soleus with endurance training by Kinnunen and Mänttäri [48], which is comparable to the gene expression results seen in our high-active mice. These observations highlight the need to use naïve animals when investigating inherent gene expression differences.

From our gene expression results we can conclude that differences in inherent variation in activity levels are not due to differences in transcript abundance of the genes investigated. Additionally, we propose that expression differences seen in *Mstn *and *Casq1* in the wheel-exposed animals did not arise through genomic structural differences. Our interstrain haplotype results indicated that five of the nine genes (*Actn2*, *Casq1*, *Drd2*, *Lepr*, and *Papss2*) contained SNPs, although none of the SNPs were located in coding regions. *Drd2* contains 5′ UTR SNPs; however, no known regulatory regions were found at these locations. None of the SNPs determined in this study were found to have obvious mechanisms of variation.

There are limitations that warrant consideration in this study, beginning with the tissues assessed, the number of strains evaluated, and the inability to compare between wheel treatments. Only slow-twitch oxidative muscle fiber was evaluated in this study without consideration of fast-twitch fibers. Previous studies [10] have shown that the average daily duration of activity in the high-active C57L/J mice was lengthy (351.1 ± 61.6 mins/day) suggesting that the slow-twitch fibers would be the primary locomotor muscles used; however, the genes we evaluated might be expressed differently in fast-twitch fibers. For instance, while Kinnunen et al. [48] found *Casq1* protein levels to decrease in soleus fibers, *Casq1* was increased in fast-twitch EDL muscle with endurance training. Additionally, while the nucleus accumbens was removed with the utmost care [26], it is possible that surrounding portions of hypothalamus were dissected along with the nucleus accumbens, leading to variability in expression levels. We do not expect this to be the case however as variability of the expression ratios of the naïve animals (as to not account for any variability caused by training adaptation) is consistent between genes in this study. Gene expression variability in the nucleus accumbens was also similar to that seen by Knab et al. [25]. Therefore, as the nucleus accumbens is considered the central reward center and a potential site of activity regulation [25, 40], we believe that our results reflect true differences in gene expression. Furthermore, it should be noted that only two strains of mice were evaluated in this study. It is possible that the mechanisms controlling activity in these two strains are specific to only those strains. While there are no direct data regarding this point, studies from our lab have shown that physical activity-QTL derived using two strain intercross methods (i.e., positional cloning approaches) [12] differs from physical activity-QTL derived using multiple strain, genome-wide association approaches [10]. Thus, it is possible that the potential candidate genes we examined in this study might be expressed differentially in other strains. Finally, it is worth mentioning that gene expression comparisons were not made between wheel-exposed and naïve animals due to variability between these groups. Given that differences between the wheel-running animals and the naïve animals were not our primary hypothesis, as well as the fact that the animals were housed at different locations (wheel-exposed animals were housed at UNC-Charlotte, while naïve animals were housed at Texas A&M) which has been known to cause different phenotypic responses [49], gene expression comparisons between wheel-exposed mice and naïve mice may possibly lead to an inaccurate depiction of the physiological differences between these groups.

In conclusion, results showed augmented gene expression of *Casq1* and *Mstn* in the soleus of low-active mice that were exposed to a running wheel. In addition, we found that exposure to a running wheel resulted in differences in transcript abundance in and of itself, implying a training effect and highlighting the need to measure gene expression in naïve mice when studying naïve genetic regulation. None of the nine suggested activity-related candidate genes were differentially expressed between inherently high- and low-active mice in soleus or nucleus accumbens. Five genes have genomic structural differences (*Actn2*, *Casq1*, *Drd2*, *Lepr,* and *Papss2*); however, no SNPs were found in coding regions nor were any associations made between any 3′ UTR SNPs and known miRNA targets. Thus, the SNPs we found do not indicate an obvious mechanism of variation. As the understanding of genetic regulation continues to mature, it is clear that considering genomic structural variation solely, as suggested by association studies, is not adequate to establish a gene's candidacy for a regulatory role and that information regarding transcriptional expression, transcriptional regulatory mechanisms, and proteomic data is needed to establish solid genetic candidates for further causal investigations.

## Figures and Tables

**Table 1:** SNP variation between high- and low-active mice.

Gene	Sequence accession no.	Chromosome no.	SNP position		Nucleotide change		Region
			On chromosome	In gene	C3H/HeJ	C57L/J	
*Actn2 *	NC_000079	1	12269977	551	T	C	intron 1
			12277437	8011	G	A	intron 1
			12282994	13568	C	T	intron 1
			12290237	20811	G	A	intron 1
			12299638	30212	A	T	intron 2
			12300030	30604	A	C	intron 2
			12301365	31939	G	A	intron 4
			12336213	66787	C	T	intron 18
			12336314	66888	G	A	intron 18

*Casq1 *	NC_000067	1	172213506	3612	T	G	intron 3
			172213681	3787	A	G	intron 3

*Drd2 *	NC_000075	9	49344757	4095	G	A	5′ UTR
			49353453	12791	A	G	5′ UTR
			49367545	26883	T	C	5′ UTR
			49372928	32266	G	A	5′ UTR
			49372964	32302	C	T	5′ UTR
			49373405	32743	A	T	5′ UTR
			49376079	35417	A	G	5′ UTR
			49376164	35502	T	C	5′ UTR
			49396183	55521	G	A	intron 1

*Lepr *	NC_000070	4	101802391	84984	C	T	intron 17
			101802709	85302	T	G	intron 17

*Papss2 *	NC_000085	19	32607198	11483	T	C	intron 1
			32607669	11954	G	A	intron 1
			32613744	18029	C	T	intron 1
			32626984	31269	C	T	intron 1
			32633562	37847	T	C	intron 1
			32649103	53388	A	C	intron 7
			32653353	57638	G	A	intron 8
			32665695	69980	C	A	3′ UTR

Haplotype and SNP data were obtained from the Mouse Phylogeny Viewer (http://msub.csbio.unc.edu/). C57L/J are high-active mice and C3H/HeJ are low-active mice.

**Table 2:** Gene expression ratios.

	Gene	Tissue	Expression ratio (AU)		*P* value
			C57L/J (high-active)	C3H/HeJ (low-active)	
Wheel exposed	*Actn2 *	sol	1.1 ± 0.32	0.95 ± 0.22	0.55
	*Casq1 *	sol	0.85 ± 0.04	1.17 ± 0.04	0.0003*
	*Glut4 *	sol	0.94 ± 0.07	1.08 ± 0.13	0.2
	*Lepr *	sol	1.0 ± 0.27	1.05 ± 0.26	0.85
	*Mstn *	sol	0.59 ± 0.05	1.7 ± 0.14	0.002*
	*Actn2 *	NA	0.14 ± 0.12	0.44 ± 0.53	0.13
	*Casq1 *	NA	0.16 ± 0.14	0.12 ± 0.05	0.64
	*Glut4 *	NA	0.17 ± 0.10	0.2 ± .07	0.58
	*Lepr *	NA	1.77 ± 2.47	1.33 ± 0.73	0.72
	*Mstn *	NA	0.12 ± 0.07	0.22 ± 0.22	0.37

Naïve	*Actn2 *	sol	1.27 ± 0.96	1.33 ± 1.1	0.58
	*Actn3 *	sol	1.25 ± 0.94	1.35 ± 1.57	0.58
	*Casq1 *	sol	1.05 ± 0.54	0.79 ± 0.40	0.4
	*Glut4 *	sol	1.22 ± 0.56	0.89 ± 0.19	0.22
	*Lepr *	sol	0.94 ± 0.66	1.03 ± 0.40	0.82
	*Mstn *	sol	0.98 ± 0.58	1.38 ± 0.55	0.27
	*Drd2 *	NA	1.69 ± 0.85	0.80 ± 0.47	0.06
	*Lepr *	NA	0.35 ± 0.09	0.26 ± 0.10	0.18
	*Mc4r *	NA	0.88 ± 0.19	1.21 ± 0.36	0.08
	*Papss2 *	NA	1.17 ± 0.33	0.97 ± 0.48	0.4

Gene expression ratio was calculated from Pfaffl [36]. Values are described as mean ± SD; “sol” indicates soleus; “NA” nucleus accumbens. *Indicates differential expression of gene between strains.
